# Plasmin inhibitor deficiency: A case report

**DOI:** 10.1002/ccr3.2624

**Published:** 2020-01-07

**Authors:** Ouissal Aissaoui, Rachid Cherkab, Lahoucine Barrou

**Affiliations:** ^1^ Pediatric ICU and Anesthesiology University Hospital of Casablanca Casablanca Morocco; ^2^ Anesthesiology and surgical ICU University Hospital of Casablanca Casablanca Morocco

**Keywords:** anesthesia, critical care medicine, emergency medicine, hematology

## Abstract

Plasmin inhibitor deficiency is an overlooked cause of hemorrhage. It is a rare disease. Delayed post‐traumatic occurrence of bleeding is an essential feature. The specific dosage must be performed to diagnose cases of severe, persistent bleeding, contrasting with normal usual tests of hemostasis.

## Graphical abstract

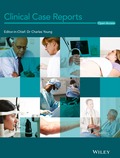

## 1 INTRODUCTION

Fibrinolysis is the set of phenomena allowing the removal of the clot inside the vessel. In hemorrhagic pathology, the plasminogen‐dependent system, specially the plasmin inhibitor deficiency, is sometimes involved.^1^ We present the observation of a 50‐year‐old patient who died of refractory hemorrhagic shock, secondary to an unknown plasmin inhibitor deficiency.

Fibrinolysis is the set of cellular and plasma mechanisms allowing the destruction of the thrombus. Its dysregulation contributes to the occurrence of thrombotic and hemorrhagic diseases. At the hemorrhagic level, the plasmin inhibitor (PI, also called α2‐antiplasmin) deficiency could be identified. It is characterized by a very low frequency and is not identified by usual or simple tests. It requires a specific dosage.^1^ This deficiency must be sought at least in well‐documented cases of persistent bleeding pathology, after the elimination of more frequent biological causes.

We report a case of a patient admitted for the management of a persistent hemorrhagic syndrome. Usual explorations of hemostasis were normal. Specific tests revealed a PI deficiency.

## 2 CASE REPORT

The patient is a 50 years old male, admitted in a state of hemorrhagic shock due to a persistant hemothorax associated with abundant hematemesis and melena. Personal history includes persistent bleeding a month after circumcision, and a peptic ulcer medically treated 4 years earlier. The patient's brother died from hemorrhagic shock, following a tooth extraction. There are no signs of hemarthrosis, hematoma, or bleeding after circumcision, nor hemophilia in the family history.

The subject was injured in a road accident one week before admission. The impact point was thoracic. The chest X‐ray was normal, and the patient received symptomatic treatment before release.

When readmitted, he was conscious, Glasgow coma scale 15/15, heart rate 136 beats per minute, blood pressure 85/40 mm Hg, respiratory rate 36, and peripheral capillary oxygen saturation SpO_2_ 85%.

The auscultation revealed an abolition of the vesicular murmur on the left. The patient was pale showing bruises and abrasions on the left hemithorax. There was no petechiae, no hepatosplenomegaly, no collateral circulation, and there was no bleeding or hematoma at the site of venous punctures.

The patient initially benefited from oxygen therapy and volume expansion with crystalloids and colloids after noninvasive monitoring of blood pressure, SpO_2_, respiratory rate, and heart rate.

The initial biological assessment showed a hemoglobin level of 7.5 g/dL and a platelet count of 189 000 elements. The hemostasis assessment was normal with prothrombin at 86%, activated partial thromboplastin time APTT at 29.8 seconds, and fibrinogen at 4 g/L. Renal function was correct with a urea level of 0.5 g/L, serum creatinine at 13 mg/L, and glomerular filtration rate GFR at 64 mL/min. There was no hepatic cytolysis, albuminemia was at 35 g/L, serum sodium level was 135 mEq/L, and potassium level was 4 mEq/L.

The patient received a red blood cells transfusion and underwent a chest drainage. Four liters was drained over 48 hours with a flow rate of 100 mL/h.

The digestive hemorrhage was persistent, so an upper gastrointestinal endoscopy was performed. It showed massive diffuse bleeding.

The hemorrhagic shock was persistent despite the blood transfusion and the administration of tranexamic acid. An exploratory laparotomy was carried out, showing a diffuse bleeding, without individualized lesion. The patient died in a state of refractory hemorrhagic shock.

We ran specific tests of hemostasis and thrombosis: The bleeding time turned out normal, the various clotting factor concentrations, including antihemophilic factors VIII and IX, von Willebrand factor, factor XIII, and factor V, were normal.

On the other hand, the dosage of plasmin inhibitor was low: 29 IU/L (normal value 80‐120 IU/L).

The diagnosis of a constitutional plasmin inhibitor deficiency was retained.

## 3 DISCUSSION

Human alpha 2‐ plasmin inhibitor « PI» is the main physiological inhibitor of the fibrinolytic enzyme plasmin. Severely reduced PI levels in hereditary PI deficiency may lead to bleeding symptoms, whereas increased PI levels have been associated with increased thrombotic risk.^2^

The constitutional PI deficiency is a rare disease.^3^ To this day, we only know of forty cases.^1^ Following a trauma or a surgery, and during the resorption of the clot, this deficiency leads to hemorrhages that can persist for several weeks. This delayed and post‐traumatic occurrence of bleeding is an essential feature, likely reflecting the delayed character of fibrinolysis.

In fact, this delayed feature is explained by the fact that primary hemostasis and coagulation are normal in these individuals. During fibrinolysis, where PI has an important role in attenuating the latter by neutralizing plasmin, the deficiency is revealed because the premature dissolution of plugs precedes the reparation of injured vessels.

In our case, the post‐circumcision bleeding persisted for one month, and the hemothorax appeared one week after the accident, announcing a defect in fibrinolysis. This symptomatology can also evoke a constitutional deficit in factor XIII,^3^ and however, the dosage of this factor was normal in our case.

Regarding the genetic basis of this deficiency, the gene coding for human PI, SERPINF2, is located on chromosome 17.^4^ Polymorphisms of the gene have also been described. The gene contains 10 exons and 9 introns, and the mature protein is encoded by exons 3‐10.^4^ The mode of inheritance is autosomal recessive,^5^ and therefore, there are differences between homozygous and heterozygous individuals.

Severe homozygous forms of PI deficiency are associated with spontaneous and, more frequently, trauma‐induced, severe bleeding.^6^ Another specific characteristic to homozygous subjects is the possibility of diaphyseal hematomas of long bones.^7^

A mild to moderate bleeding diathesis has been reported in subjects heterozygous for PI deficiency.^6^ These subjects can even be asymptomatic.^7^ They may only be revealed after surgery. The intensity of the manifestations may increase with age. Biological diagnosis is usually a diagnosis of exclusion, sometimes difficult to achieve. Indeed, these patients are repeatedly investigated for an etiology to an unusual hemorrhagic syndrome, by conventional tests of hemostasis that remain normal (time of quick, activated partial thromboplastin time, thrombin time, dosage of fibrinogen, platelet aggregation, von Willebrand factor,…). The detection of the deficit requires the specific dosage of PI, which is not often performed given the scarcity of occurrences and can lead to absence of diagnosis.^8^ Inhibitory activity is quantified using a chromogenic substrate sensitive to plasmin.^9^ Activation of the reaction is carried out by the addition of plasmin. Residual plasmin is measured after the action of the PI present in the plasma.^9^

A characterization of the type of deficit can be achieved by measuring the antigen. The deficit is then classified as type I or type II depending on the absence or the presence of a molecule recognized immunologically and with low activity.^1^

Therapeutic management may be limited to the use of antifibrinolytics such as tranexamic acid.^10^ This measure was insufficient in our case. Fresh frozen plasma containing functional PI may be required.^1^

## 4 CONCLUSION

Fibrinolysis is the set of cellular and plasma mechanisms allowing the destruction of the thrombus. Its dysregulation contributes to the occurrence of thrombotic and hemorrhagic diseases. At the hemorrhagic level, PI deficiency is a rare, unusual, and difficult diagnosis. It must be sought in case of delayed hemorrhagic syndrome, contrasting with normal tests of hemostasis.

## CONFLICTS OF INTEREST

The authors declare that there is no conflict of interest.

## AUTHOR CONTRIBUTIONS

Ouissal Aissaoui: contributed to drafting of manuscript. Rachid Cherkab: contributed to critical revision. Lahoucine Barrou: contributed to conception and final approval.
